# EMCC dispatch priority for trauma patients in Norway: a retrospective cohort study

**DOI:** 10.1186/s13049-025-01387-2

**Published:** 2025-05-12

**Authors:** Inger Marie Waal Nilsbakken, Torben Wisborg, Stephen Sollid, Elisabeth Jeppesen

**Affiliations:** 1 Department of Research, Norwegian Air Ambulance Foundation, Postboks 414 Sentrum, 0103 Oslo, Norway; 2 Faculty of Health Sciences, University of Stavanger, Stavanger, Norway; 3 Interprofessional rural research team – Finnmark, Faculty of Health Sciences, University of Tromsø – the Arctic University of Norway, Tromsö, Norway; 4 Norwegian National Advisory Unit on Trauma, Division of Emergencies and Critical Care, Oslo University Hospital, Oslo, Norway; 5 Faculty of Health Studies, VID Specialized University, Oslo, Norway

**Keywords:** Trauma, Prehospital care, Dispatch priority, Emergency medicine, Trauma registry

## Abstract

**Background:**

Dispatch priority assessments in emergency medical communication centres (EMCC) play a crucial role in determining how quickly emergency medical services reach the scene after an injury. Consequently, accurate prioritization of resources is important in ensuring that patients requiring specialized care receive timely treatment to optimize their outcome. Both dispatch under-triage, where patients with severe injuries receive low priority, and dispatch over-triage, which unnecessarily allocates limited emergency resources, can impact patient outcomes and system efficiency. This study aimed to assess dispatch priority in the EMCC for a cohort of trauma patients in Norway.

**Methods:**

This registry-based study included 3633 patients from the Norwegian Trauma Registry and Oslo EMCC during 2019–2020. We assessed sensitivity, specificity, positive predictive value (PPV), negative predictive value (NPV), false negative rate (dispatch under-triage rate), false positive rate (dispatch over-triage rate), and accuracy of dispatch priority. The New Injury Severity Score (NISS) > 15 was used as a reference standard. Differences in dispatch priority assessments were analysed using descriptive statistics. Two logistic regression models were used to examine the relationship between dispatch priority and factors associated with the assessment.

**Results:**

Our analysis revealed the following dispatch metrics: sensitivity (85%), specificity (11%), PPV (38%), NPV (53%), dispatch under-triage rate (15%), dispatch over-triage rate (89%), and overall accuracy (40%). Under-triaged dispatches frequently involved elderly trauma patients (53%) and patients with low-energy falls (51%). Elderly trauma patients had more than 7 times the odds of receiving inappropriately low dispatch priority compared to children and nearly twice the odds compared to adults, after accounting for factors such as injury mechanism. Similarly, female patients had 81% higher odds of receiving inappropriately low dispatch priority compared to male patients, when controlling for factors like age and injury mechanism. Among over-triaged dispatches, transport-related injuries accounted for half of the cases (50%).

**Conclusion:**

This study primarily evaluated the national trauma system’s dispatch priority criteria. Our findings indicate that elderly trauma patients, those with low-energy falls and female patients were often assigned inadequate priority by current criteria, indicating a need to reassess the current criteria to better address these patients’ needs. Additionally, we found that patients involved in transport-related accidents were overrepresented among over-triaged dispatches, highlighting a potential misallocation of resources.

## Background

A core component of trauma systems is delivering the right treatment to the right patient at the right time. For trauma patients requiring specialized care, timely intervention by emergency medical services (EMS) can be important to improve outcomes [1]. Dispatch priority assessments affect EMS response times, making correct prioritization essential to minimize delays. Dispatch over-triage, or the unnecessary allocation of limited EMS resources on patients with minor injuries, can strain prehospital healthcare systems, while dispatch under-triage, failing to recognize major trauma, may lead to suboptimal care and worse outcomes [2]. Appropriate allocation of finite resources is particularly important in countries like Norway, where long distances between people and hospitals, variable infrastructure and rough seasonal weather challenge EMS availability [3].

Few studies have investigated dispatch priority assessments and factors associated with inappropriate prioritization in emergency medical communication centres (EMCC). The available research indicates that most patients with high-priority needs receive the appropriate priority [4, 5]. Waalwiljk et al. found that 82.5% of patients with an Injury Severity score (ISS) > 15 were assigned the highest dispatch priority. Salminen et al. investigated the priority differences between EMCC operators and EMS professionals and found that 3% of non-urgent dispatches by EMCC operators were in fact urgent according to EMS criteria. However, 71% of urgent dispatches by EMCC operators were non-urgent according to the EMS criteria. This indicates that many patients were assigned high priority, but did not actually require it [5].

Research also indicates that factors such as gender and age may influence priority assessments [4, 5]. However, which factors in general impact the priority assessment is largely unknown, particularly regarding the magnitude of each factor and, not least, how different factors interact. Although many unquantifiable factors contribute to EMCC priority assessments, there are measurable factors known at the time of assessment. The purpose of this study was to assess dispatch priority and factors associated with inappropriate prioritization in a cohort of trauma patients in Norway. It primarily evaluated the national trauma system’s criteria for priority assessment (the Norwegian Index for Medical Emergency Assistance). To our knowledge, no prior research has explored dispatch priority in the Norwegian EMCC in such depth, nor investigated the factors contributing to correct or incorrect prioritization.

## Methods

### Study design

This was a registry-based retrospective observational study using data from the National Trauma Registry (NTR) and Oslo EMCC from 1st of January 2019 to 31st of December 2020.

### Setting

The study population is comprised of residents in the south-east of Norway covered by the Oslo EMCC. The Oslo EMCC serves most of the south-east health region, covering the admission areas of Oslo University Hospital, Akershus University Hospital, Østfold Hospital and the municipalities of Asker and Bærum, in addition to regional coordination tasks for the south-east health authority [6, 7]. This area has an estimated population of 2 million people, about one-third of the Norwegian population [8]. Norway has a publicly funded healthcare system with four regional health authorities and a national trauma system, which includes trauma centres (TC) in each regional health area and acute care trauma hospitals (ACTH) designated to receive and treat trauma patients.

### Outcomes and definitions

The main objective was to assess the sensitivity, specificity, positive predictive value (PPV), negative predictive value (NPV), false negative rate (FNR), false positive rate (FPR), and accuracy of dispatch priority assessments in the EMCC in Norway.

- Sensitivity: ability to correctly identify severe injuries $\to$ high dispatch priority for patients with a New Injury Severity Score (NISS) > 15.
- Specificity: ability to correctly identify minor injuries $\to$ low dispatch priority for patients with NISS < 9.
- PPV: probability that a high-priority patient truly had a severe injury.
- NPV: probability that a low-priority patient truly did not have a severe injury.
- FNR (dispatch under-triage rate): the proportion of patients with NISS > 15 who were assigned low dispatch priority.
- FPR (dispatch over-triage rate): the proportion of patients with NISS < 9 who were assigned high dispatch priority.
- Accuracy: overall correctness of the priority classification.

The second objective was to identify factors associated with inappropriate dispatch allocation (i.e. dispatch under-triage and dispatch over-triage). In this we included objective, measurable factors that were known at the time of EMCC dispatch, including age, gender, consciousness level and injury mechanism.

Patients with moderate injury (NISS 9–15) were considered intermediate cases, where the need for high priority is difficult to determine.

### Data sources and study sample

Data were collected from the NTR and the Oslo EMCC activity database (AMIS). The NTR is a national clinical quality registry containing data on all injured patients in Norway (based on the Utstein template [9]). The social security numbers of patients registered in the NTR were used to identify the corresponding dispatch priority assessment in the AMIS database. Most trauma patients transported to hospitals by EMS are registered in both systems and NTR registrars use information from the EMCC AMIS system to complete NTR entries. Table 1 lists the inclusion criteria for the NTR. The study included patients recorded in both the NTR and AMIS at Oslo EMCC by social security number. Only first hospital assignments were included (transfers were excluded), and patients with trauma team activation but no trauma (i.e., medical conditions) were excluded.

**Table 1:** Inclusion criteria for the Norwegian trauma registry (NTR)

**Inclusion** All patients admitted with trauma team activation (TTA) upon arrival to the emergency department in all acute care trauma hospitals and trauma centres in Norway, irrespective of injury severity All patients treated at an acute care trauma hospital or trauma centre in Norway, without TTA, with one or more or the following criteria/injuries: • Penetrating injury to the head, neck, torso or extremities proximal to elbow or knee • Head injury with abbreviated injury score (AIS) ≥ 3 • New Injury Severity Score (NISS) > 12 All patients who suffered trauma-related deaths at site of trauma or during transportation to hospital, who were not referred to hospital, but where prehospital management was initiated	

### Dispatch priority

In Norway, medical emergencies have a dedicated emergency number (113), distinct from police and fire services. All calls are assessed by EMCC operators who are nurses or paramedics with additional training. The EMCC operators handle the initial emergency call, determine whether the patient needs on-site evaluation by an EMS professional, and assign the appropriate dispatch priority for the EMS request (acute, urgent and normal). The operators use the Norwegian Index for Medical Emergency Assistance (Index) [10] to identify time-critical conditions, including trauma cases, to determine the dispatch priority. This tool, developed by the Norwegian Medical Association, is the standard used for dispatch priority assessment. The dispatch priorities include acute, urgent and normal. For acute dispatch priority, the patient has a manifest or threatening failure of vital functions, triggering an immediate EMS response. For urgent dispatch priority, the patient requires medical assessment to clarify the potential for vital function failure, allowing more time for EMS resources to be dispatched [11].

The EMCC operator typically dispatches ground EMS units, which may include varying levels of staffing (ambulance workers and/or paramedics). For cases requiring additional medical expertise, the EMCC operators can dispatch primary care physicians from local casualty clinics, who often respond together with ground EMS units or in their own emergency vehicle often together with an emergency nurse. For more severe cases, the EMCC operator may also activate the Helicopter Emergency Medical Service (HEMS), which provides rapid transport and advanced medical care through teams that include a pilot, a rescuer/paramedic, and an anaesthesiologist. The HEMS crews have rapid response vehicles available for deployment in urban areas where helicopter landing may be impractical or when weather conditions restrict flying. In the Oslo area, there are dedicated emergency response vehicles staffed with an anaesthesiologist and a paramedic who can provide advanced medical interventions at the scene. In some situations, multiple resources may be deployed simultaneously to ensure comprehensive prehospital care.

Indicative response times for EMS (the time from when the EMCC operator is notified until EMS arrives at the scene) have been established by Norwegian authorities. For acute dispatch priority, EMS should arrive at the scene within 12 min in 90% of cases in urban and suburban areas, and within 25 min in 90% of cases in remote areas. For urgent dispatch priority, EMS should arrive within 30 min in 90% of cases in urban and suburban areas, and within 40 min in 90% of cases in remote areas [12]. These indicative response times illustrate the differences between the priorities acute and urgent. Additionally, national trauma criteria for suspected severe injury have also been developed and implemented [13].

### Variables

Dispatch priority (acute, urgent and normal) was collected from the AMIS database at Oslo EMCC. As only 1% of trauma patients were assigned ‘normal’ priority, we reduced the number of priority groups from 3 to 2 by merging ‘urgent’ and ‘normal’ into ‘low priority’. Dispatch priority assessment ‘acute’ was renamed ‘high priority’. Data variables collected from the NTR include age, gender, injury mechanisms, injury severity (according to NISS), prehospital Glasgow Coma Scale (GCS) score for prehospital level of consciousness and the accident municipality for Centrality Index calculation. The continuous age variable was grouped into three categories: ‘children (0–15 years)’, ‘adults (16–66 years)’ and ‘the elderly (> 66 years)’. Some injury mechanisms were re-categorized from the original NTR definitions: five traffic-related injury mechanisms (motor vehicle, motorcycle, bicycle, pedestrian and other) were merged into ‘transport-related’, while ‘shot by firearm’, ‘explosion injury’ and ‘other’ were merged into ‘other’. GCS scores of 14 or 15 were merged into one category (‘prehospital GCS 14–15’), and scores below 14 were merged into another (‘prehospital GCS < 14’).

### Centrality index

The Centrality Index (CI) measures a municipality’s centrality based on travel time to workplaces and services, and the proportion of inhabitants [14, 15]. Municipalities are classified into six categories. However, due to the low number of trauma patients in remote municipalities, we reduced the categories from six to three by merging neighbouring index groups: CI groups 1 and 2 became ‘urban areas’, groups 3 and 4 became ‘suburban areas’ and groups 5 and 6 became ‘remote areas’.

### Statistical methods

We analysed the registry data using descriptive statistics to summarize the data, with categorical variables presented as frequencies and percentages, and continuous variables as means with standard deviations (SD) or medians with interquartile ranges (IQR), as appropriate. Sensitivity, specificity, PPV, NPV, FNR, FPR, and overall accuracy were calculated to evaluate classification performance. A Student’s t-test was used to assess differences in age means across regions (urban, suburban, and remote). A Kruskal–Wallis test was conducted to assess differences in NISS medians across regions. Several chi-square tests of independence were performed to assess differences in injury mechanisms, age groups and gender across the regions. To assess factors associated with dispatch under-triage and dispatch over-triage, we used two binary multivariate logistic regression models: one for patients with NISS > 15 and one for patients with NISS < 9. In these models, dispatch triage was the dependent variable, while age groups, injury mechanism, gender, prehospital GCS score, NISS and CI were independent variables. Results from these models were presented as odds ratios (OR) with 95% confidence intervals and p-values. The likelihood ratio test (LRT) was used to assess whether adding independent variables to the model significantly improved the model fit. Nagelkerke’s R^2^ was used to approximate the proportion of variance explained by the model and to assess model fit alongside LRT. Multicollinearity between predictor variables was evaluated using variance inflation factors (VIF), with a threshold of VIF > 10 indicating potential multicollinearity. A p-value of ≤ 0.05 was considered statistically significant. All analyses were conducted using SPSS v. 27.0 (IBM Company, Chicago, IL, USA).

## Results

A total of 4842 trauma patients were registered in the NTR and Oslo EMCC AMIS database during the study period. Of these, 1209 were transfers, resulting in 3633 trauma patients included in the study. Sixty-five per cent of these patients were injured in urban areas, 26% in suburban areas and 9% in remote areas.

### Study population characteristics

Study population characteristics are presented in Table 2. There were significant differences in injury mechanism distribution, mean NISS, age groups and gender distribution between regions (urban, suburban, and remote). Statistical testing did not reveal any significant differences in mean age between regions.

**Table 2:** Study population characteristics

Variables		All	Urban	Suburban	Remote	*p*-value
Age groups						0.005^a^
0–15	Per cent	461 (13%)	308 (13%)	101 (11%)	52 (16%)	
16–66	Per cent	2407 (68%)	1542 (66%)	642 (69%)	223 (70%)	
> 66	Per cent	698 (19%)	475 (20%)	181 (20%)	42 (13%)	
Age	Mean (SD)	43 (24)	44 (24)	43 (24)	41 (22)	ns^b^
Male	Per cent	2384 (67%)	1520 (65%)	645 (70%)	219 (69%)	0.04^a^
NISS groups						< 0.001^a^
< 9	Per cent	1584 (48%)	1121 (52%)	354 (42%)	109 (36%)	
9–15	Per cent	725 (22%)	464 (21%)	176 (21%)	85 (28%)	
> 15	Per cent	1016 (30%)	588 (27%)	321 (38%)	107 (36%)	
NISS	Median (IQR)	9 (15)	6 (15)	10 (15)	11 (13.5)	< 0.001^c^
Dominant injury						
Blunt	Per cent	3346 (94%)	2163 (94%)	879 (95%)	304 (96%)	ns^a^
Injury mechanism						< 0.001^a^
Transport-related	Per cent	1597 (46%)	1012 (44%)	449 (49%)	136 (44%)	
Low-energy fall	Per cent	566 (16%)	421 (18%)	113 (12%)	32 (10%)	
High-energy fall	Per cent	762 (21%)	461 (20%)	202 (22%)	99 (32%)	
Being struck or hit by blunt object	Per cent	347 (10%)	237 (10%)	88 (10%)	22 (7%)	
Being stabbed by a knife or sharp object	Per cent	154 (4%)	125 (5%)	23 (2%)	6 (2%)	
Other	Per cent	119 (3%)	57 (2%)	46 (5%)	16 (5%)	

*SD* standard deviation, *ns* non-significant, *NISS* New Injury Severity Score, *IQR* interquartile range

^a^Chi square test of independence

^b^Student’s t-test

^c^Kruskal-Wallis test

Table 3 presents the distribution of cases assigned low or high dispatch priority, categorized according to the level of treatment provided by the prehospital crew, which includes different levels of care such as basic life support and advanced life support led either by paramedic/ambulance crew or physician.

**Table 3:** Counts and proportions of low and high dispatch priority cases by level of treatment provided by the prehospital crew

	BLS or ALS led by paramedic or ambulance crew	ALS led by physician
Low dispatch priority	468 (97%)	17 (3%)
High dispatch priority	2372 (77%)	716 (23%)

*BLS* basic life support, *ALS* advanced life support

### Dispatch priority

Table 4 lists the breakdown of patients with injury severity (NISS) and dispatch priority assessment where true positives, false negatives, false positives and true negatives were identified. From these values we calculated the dispatch sensitivity, specificity, PPV, NPV, FNR, FPR, and overall accuracy found in Table 5.

**Table 4:** Breakdown of patients with injury severity (NISS) and dispatch priority assessment

	NISS > 15	NISS < 9
High dispatch priority	872 (TP)	1441 (FP)
Low dispatch priority	156 (FN)	175 (TN)

*TP* true positives, *FN* false negatives, *FP* false positives, *TN* true negatives

**Table 5:** Calculations of dispatch sensitivity, specificity, PPV, NPV, FNR, FPR and accuracy

Parameter	Formula	n	Value	Per cent
Sensitivity	$\frac{TP}{{TP + FN}}$	$\frac{872}{{872 + 156}}$	0.85	85
Specificity	$\frac{TN}{{TN + FP}}$	$\frac{175}{{175 + 1441}}$	0.11	11
PPV	$\frac{TP}{{TP + FP}}$	$\frac{872}{{872 + 1441}}$	0.38	38
NPV	$\frac{TN}{{TN + FN}}$	$\frac{175}{{175 + 156}}$	0.53	53
FNR	1—sensitivity	1—0.85	0.15	15
FPR	1—specificity	1—0.11	0.89	89
Accuracy	$\frac{TP + TN}{{TP + TN + FP + FN}}$	$\frac{872 + 175}{{872 + 175 + 1441 + 156}}$	0.40	40

*PPV* positive predictive value, *NPV* negative predictive value, *FNR* false negative rate, *FPR* false positive rate

### Factors associated with inappropriate dispatch allocation

Table 6 lists the characteristics of patients with dispatch under-triage.

**Table 6:** Characteristics of patients with dispatch under-triage

Variables		
Male	Count (per cent)	84 (54%)
Age	Mean (SD)	65 (21)
Age groups		
0–15	Count (per cent)	< 5 (2%)
16–66	Count (per cent)	70 (45%)
> 66	Count (per cent)	83 (53%)
NISS	Median (IQR)	19 (9.75)
Injury mechanism distribution		
Transport-related	Count (per cent)	28 (18%)
Low-energy fall	Count (per cent)	79 (51%)
High-energy fall	Count (per cent)	36 (23%)
Being struck or hit by blunt object	Count (per cent)	8 (5%)
Being stabbed by a knife or sharp object	Count (per cent)	< 5 (1%)
Other	Count (per cent)	< 5 (1%)

Among patients with low dispatch priority and low-energy falls, the majority (79%) were elderly.

A multivariate logistic regression model was performed with age groups, gender, injury mechanisms, prehospital consciousness level (prehospital GCS), NISS and CI as independent variables, and dispatch priority as the dependent variable for patients with NISS > 15, to assess factors associated with dispatch under-triage.

Holding other independent variables constant, we found that children and adult patients were associated with lower odds of low dispatch priority compared with elderly patients (odds ratios (OR) 0.13 and 0.59, p-values 0.009 and 0.04, respectively). The inverse of these odds ratios is equal to 7.69 and 1.69, meaning that elderly patients had higher odds of low dispatch priority compared with children and adults. Patients with low-energy and high-energy falls were associated with higher odds of low dispatch priority compared with patients with transport-related injuries (OR 7.4 and 2.02, *p*-values < 0.001 and 0.03, respectively). We found that female patients were associated with 81% higher odds of low dispatch priority compared with male patients (OR 1.81, *p*-value 0.02). A high prehospital consciousness level (prehospital GCS 14–15) was associated with higher odds of low dispatch priority compared with patients with low prehospital consciousness levels (prehospital GCS < 14) (OR 5.43, *p*-value < 0.001). The centrality index variable as a whole was not a statistically significant predictor, but the specific comparisons between the different centrality index areas revealed significant results, where patients in both urban and suburban areas were associated with higher odds of low dispatch priority compared with patients in remote areas (OR 3.1 and 3.52, *p*-values 0.04 and 0.03, respectively) (Table 7). These results reflect the results of a model that adjusted for all other independent variables in the analysis.

**Table 7:** Factors associated with dispatch under-triage

Independent variables	B	OR	95% CI OR	p-value
Age groups				0.008
Children	− 2.01	0.13	0.03–0.60	0.009
Adults	− 0.53	0.59	0.35–0.98	0.04
The elderly	Ref.			
Gender				
Male	Ref.			
Female	0.60	1.81	1.12–2.92	0.02
Injury mechanisms				< 0.001
Transport-related	Ref.			
Low-energy falls	2.00	7.4	3.92–13.89	< 0.001
High-energy falls	0.70	2.02	1.08–3.77	0.03
Being struck or hit by blunt object	− 0.03	0.97	0.27–3.47	0.96
Being stabbed by a knife or sharp object	0.80	2.18	0.44–10.90	0.33
Other	0.78	2.18	0.44–10.80	0.34
Prehospital GCS				
< 14	Ref.			
14–15	1.69	5.43	2.32–12.68	< 0.001
Centrality Index				0.09
Urban	1.12	3.1	1.03–9.12	0.04
Suburban	1.26	3.52	1.16–10.66	0.03
Remote	Ref.			
NISS	− 0.03	0.97	0.94–1.0	0.06

*OR* odds ratios, *CI* confidence interval, *GCS* Glasgow Coma Score

Nagelkerke R square = 0.31

Table 8 lists the characteristics of patients with dispatch over-triage.

**Table 8:** Characteristics of patients with dispatch over-triage

Variables		
Male	Count (per cent)	954 (66%)
Age	Mean (SD)	35 (22)
Age groups		
0–15	Count (per cent)	260 (18%)
16–66	Count (per cent)	1037 (72%)
> 66	Count (per cent)	144 (10%)
NISS	Median (IQR)	2 (3)
Injury mechanism distribution		
Transport-related	Count (per cent)	720 (50%)
Low-energy fall	Count (per cent)	133 (9%)
High-energy fall	Count (per cent)	261 (18%)
Being struck or hit by blunt object	Count (per cent)	169 (12%)
Being stabbed by a knife or sharp object	Count (per cent)	100 (7%)
Other	Count (per cent)	54 (4%)

A multivariate logistic regression model was performed with age groups, gender, injury mechanisms, prehospital consciousness level (prehospital GCS), NISS and CI as independent variables, and dispatch priority as the dependent variable for patients with NISS < 9, to assess factors associated with dispatch over-triage.

Holding other independent variables constant, we found that patients with injury mechanisms such as low-energy falls, high-energy falls, being struck or hit by a blunt object and other were associated with lower odds of high dispatch priority compared with patients with transport-related injuries (OR 0.13, 0.31, 0.28 and 0.27, *p*-values < 0.001, < 0.001, < 0.001 and 0.004, respectively). The inverse of these odds ratios is equal to 7.69, 3.23, 3.57 and 3.70, meaning patients with transport-related injuries had higher odds of high dispatch priority compared with those with low-energy falls, high-energy falls, blunt object injuries and other injuries. The centrality index variable as a whole was a statistically significant predictor, and the specific comparisons between the different centrality index areas revealed significant results, where patients in urban areas were associated with lower odds of high dispatch priority compared with those in remote areas (OR 0.23, *p*-value 0.01). The inverse of this odds ratio is equal to 4.35, meaning that patients in remote areas had higher odds of high dispatch priority compared with those in urban areas (Table 9). These results reflect the results of a model that adjusted for all other independent variables in the analysis.

**Table 9:** Factors associated with dispatch over-triage

Independent variables	B	OR	95% CI OR	*p*-value
Age groups				0.61
Children	0.20	1.22	0.63–2.38	0.55
Adults	0.22	1.25	0.71–2.18	0.44
The elderly	Ref.			
Gender				
Male	0.03	1.03	0.69–1.53	0.90
Female	Ref.			
Injury mechanisms				< 0.001
Transport-related	Ref.			
Low-energy falls	− 2.02	0.13	0.08–0.24	< 0.001
High-energy falls	− 1.18	0.31	0.18–0.53	< 0.001
Being struck or hit by blunt object	− 1.27	0.28	0.15–0.52	< 0.001
Being stabbed by a knife or sharp object	− 0.06	0.94	0.32–2.77	0.92
Other	− 1.32	0.27	0.11–0.65	0.004
Prehospital GCS				
< 14	0.41	1.50	0.86–2.61	0.15
14–15	Ref			
Centrality Index				0.009
Urban	− 1.48	0.23	0.07–0.74	0.01
Suburban	− 0.92	0.40	0.11–1.40	0.15
Remote	Ref			
NISS	− 0.05	0.96	0.86 – 1.06	0.39

*OR* odds ratios, *CI* confidence interval, *GCS* Glasgow Coma Score

Nagelkerke R square = 0.12

## Discussion

Our findings reveal four key aspects of dispatch priority in trauma care that will be further discussed below. First, the majority of patients in need of high dispatch priority were correctly assigned high priority. Second, elderly trauma patients had higher odds of being inappropriately assigned low dispatch priority compared to children and adults. Third, female patients had higher odds of being inappropriately assigned low dispatch priority compared to male patients. Fourth, a large proportion of patients who did not require high priority were assigned high priority, indicating potential overtreatment.

It is important to note that the majority of patients in need of high dispatch priority were assigned high priority. We used patients with NISS > 15 (severe injury) to determine which patients required high dispatch priority. We found that 85% of patients with NISS > 15 were assigned high dispatch priority, resulting in a dispatch sensitivity of 85%. Our results are comparable to a study on dispatch priority in trauma patients in the Netherlands, where Waalwijk et al. investigated the sensitivity of dispatch priority, defined as the proportion of patients in need of specialized care who were assigned the highest dispatch priority. Patients were considered in need of specialized care if they were admitted to an intensive care unit, underwent an emergency intervention within 24 h, died within 24 h after admission or were prehospitally intubated. The authors also used ISS > 15 as a reference standard for determining the need for specialized care. They found a dispatch sensitivity of 83.8%, and among patients with ISS > 15, a dispatch sensitivity of 82.5% [4].

In trauma care, the concept of triage is important for resource allocation and patient outcomes [2]. Dispatch triage in particular serves as the first decision point that determines the level of prehospital care provided. Our findings on dispatch sensitivity (85%) should be interpreted within the broader context of acceptable under-triage rates. The American College of Surgeons Committee on Trauma (ACS-COT) recommends an under-triage rate of less than 5% and an over-triage rate of 25–35% for optimal trauma system performance [2]. It's important to note that these recommendations primarily address hospital-based trauma team activation rather than whether trauma patients received appropriate prehospital response based on injury severity. Despite these methodological differences, the conceptual framework of balancing resource allocation remains relevant. Our dispatch under-triage rate of 15% suggests potential room for improvement in identifying severely injured patients at the dispatch stage, even when accounting for the different context of measurement. The corresponding negative predictive value of 53% indicates that just over half of the patients assigned low priority did not have severe injuries.

The consequences of dispatch under-triage extend beyond simple statistical observations. When severely injured patients are not assigned high priority, they may experience delayed access to appropriate prehospital care which can potentially lead to increased morbidity and mortality, particularly in time-sensitive conditions such as traumatic brain injury and haemorrhage. Our results show effective resource allocation differences, with only 3% of low-priority dispatches receiving physician-led advanced life support (ALS), compared to 23% of high-priority dispatches. The vast majority (97%) of low-priority dispatches were managed by ambulance workers or paramedics providing BLS or ALS. This treatment level difference highlights the practical impact of triage decisions on the clinical resources available to trauma patients. Notably, the 23% physician presence rate for high-priority dispatches represents good coverage considering our study was conducted in and around Oslo, where distances to hospitals are generally short, potentially reducing the perceived need for on-site physician intervention in some high-priority cases.

Previous research has shown that under-triaged trauma patients have higher mortality rates compared to appropriately triaged patients [14, 15] and that elderly patients are at higher risk of being undertriaged [16]. Among the under-triaged patients in our study, we found an overrepresentation of elderly trauma patients (53%). Also, in our regression model, adjusted for control variables such as gender, injury mechanism, injury severity and prehospital consciousness level, elderly trauma patients had more than 7 times the odds of being inappropriately assigned a low dispatch priority compared with children and nearly twice the odds compared with adults. In Waalwijk et al.’s study on priority assessment in trauma patients in the Netherlands [4], they found that patients who received higher priority had a mean age of 48 years, while those who received lower priority had a mean age of 68 years, indicating the importance of age in priority assessments in other trauma systems as well. Furthermore, a previous study on the Norwegian trauma population [17] found that elderly trauma patients received fewer prehospital resources compared to adult patients and had lower rates of trauma team activation, despite a higher proportion of patients with NISS > 15. The predominant mechanism of injury among the elderly patients in Cuevas-Østrem et al.’s study was low-energy falls, and the mortality rate among the elderly was significantly higher (13%) compared with younger adults (2.9%) [17]. While our study did not assess mortality, we found that low-energy falls were associated with dispatch under-triage, and that 79% of these patients were the elderly. Acute illness and injury in the elderly tends to manifest differently than in younger adults, making it challenging to recognize severe injury in this age group. Falls from one's own height are a common cause of severe injury in elderly, possibly due to comorbidities, fragility and medications [18]. Moreover, as the global population ages, the number of elderly trauma patients is expected to rise [19]. For elderly patients, the consequences of dispatch under-triage may be more pronounced due to reduced physiological reserve and comorbidities that can mask or complicate injury presentations. Other studies have shown that elderly trauma patients are particularly vulnerable in the early prehospital phase and are more likely to experience poor outcomes [16, 20]. Revising dispatch priority criteria to better capture this patient group is important for improving trauma care in Norway. The National Centre for Prehospital Emergency Medicine is currently revising the Index, with input from the National Centre for Emergency Primary Health Care regarding elderly patients [21]. The goal is to develop a decision-support tool to ensure that injury is detected and that priority assessment and treatment for this growing group are improved and correct.

In addition to age-related differences, we found that female patients had 81% higher odds of inappropriately low dispatch priority compared with male patients, adjusted for injury mechanism, injury severity, age and prehospital GCS. These results are comparable to previous research on gender differences in access to services and scene-to-hospital priority, both in medical emergencies and trauma [4, 22, 23]. Wahlin et al. found in a Swedish trauma system study that males had 2.75 times higher odds of receiving higher prehospital priority compared with females, controlling for injury mechanism and vital signs at the scene, and that males were more likely to be directed to a trauma centre (OR 1.36). However, they found no gender differences in mortality [22]. The reasons for these differences are unclear. One possible factor is that female trauma patients may exhibit different symptoms, which can complicate the assessment of injury severity and hinder correct prioritization. Mellum et al. [24] conducted a qualitative study on EMCC operators in Norway, investigating gender differences in trauma patient prioritization. In focus groups and semi-structured interviews, operators stated that emergency medical responses were largely based on national trauma criteria, with gender having little or no influence during dispatch. The authors concluded that gender differences in emergency medical response appear to be caused by factors other than those considered during the emergency medical response phase, and they questioned whether trauma criteria effectively detect severe injury in women [24].

We found a dispatch specificity of 11%, meaning that the criteria correctly identified just over one-tenth of patients who did not require high priority. Our dispatch over-triage rate of 89% aligns with Salminen et al.’s findings from their study on the Finnish trauma system, where most (71%) of patients with high priority dispatch did not actually require it [5]. The positive predictive value of 38% further illustrates this issue, showing that only about one-third of patients receiving high priority actually needed it. In our study, the majority of inappropriately high priority assessments were for transport-related injuries. This may be a holdover from the past, when traffic accidents, often with high energy involved, were associated with major injuries. Overtreatment is therefore a concern, particularly for patients with minor injuries from transport-related incidents who might receive priority beyond what is required. While our focus on dispatch under-triage is justified by direct patient safety concerns, the high dispatch over-triage rate of 89% also warrants serious consideration from a system perspective. Dispatch over-triage places significant strain on limited emergency resources, potentially leading to unavailability of resources for patients who truly need them, contributing to system-inefficiency. Moreover, the financial cost of over-triage in trauma systems can be substantial [25]. However, finding the optimal balance between under-triage and over-triage remains challenging, as efforts to reduce one typically increase the other.

Finally, the overall accuracy of the dispatch priority assessment was 40%, reflecting the combined effect of good sensitivity but poor specificity. This highlights the need to improve correct identification of both high and low priority patients, particularly among vulnerable groups.

## Limitations

In large registry studies, there is a risk of type 1 errors. Moreover, as with other registry-based research, there is also a possibility of selection bias. To minimize this, our analysis included particularly relevant covariates to help control for this effect [26]. This is especially relevant in our regression model, where the main goal was to identify statistical relationships between factors and outcomes. Our results are based on data from only one EMCC in Norway, from the most central areas in the country where the hospital density is high and where the proportion of remote trauma patients is small. Differences in the assessment of dispatch priority may occur between different EMCCs in Norway, although they all use the same framework for prioritization (Index).

In our study we have used NISS > 15 as the threshold for injury severity. The NISS is a well-established method for assessing injury severity, however, alternative grading systems also exist (Need for Trauma Intervention (NFTI)) [27]. The choice of severity scoring method may influence the results, and different tools could potentially result in varying findings.

Another limitation is that not all hospitals reporting data to the NTR actively search for undertriage (patients with NISS > 15 who were not received by the trauma team). Only hospitals where registrars actively search for patients with ISS/NISS > 15 who were not received by trauma teams, include these undertriaged patients in the NTR. During the study period, two smaller ACTHs in the Oslo EMCC’s admission area did not search for undertriage. This means there may be a few patients from the period who were not included in the study. However, the majority of patients are treated at hospitals that actively monitor undertriage, and the authors believe this is representative of the hospitals that receive the most trauma patients in the region.

The sensitivity, specificity, PPV, NPV, FNR, FPR and accuracy are calculated based on the included sample of NISS groups. By excluding intermediate cases (NISS 9 – 15), these metrics may be slightly over- or underestimated compared to a real-world setting where intermediate cases exist. However, in our material only approximately 20% of cases were intermediate, and we believe our results reflect the performance of dispatch triage, especially regarding the prioritization of vulnerable groups and overutilization of resources on transport-related injuries.

The variable completeness in our study is high (≥ 85%), though 7% of patients lacked NISS, excluding it from prioritization assessment. The NTR maintains high variable completeness with 100% for quality indicators. A 2021/2022 validation showed 100% coverage for trauma team patients and 92.2% overall coverage. The 2022/2023 validation found good accuracy with 48/59 variables exceeding 90% accuracy. AIS isn't directly recorded in medical records but requires interpretation from documentation (patient journal record and radiology descriptions) [28]. The interpretive process required for AIS registration may explain why NISS is occasionally missing, as NISS is calculated directly from AIS scores.

## Conclusion

This study primarily evaluated the national trauma system’s criteria for dispatch priority assessment. Our findings revealed that elderly trauma patients, patients with low-energy falls and female trauma patients were often inadequately captured by the current criteria, indicating a need to reassess the criteria to better address these patients’ needs. Additionally, we found that patients involved in transport-related accidents were overrepresented among those assigned high-priority dispatch, despite not requiring such prioritization, highlighting a potential misallocation of resources. Future research should focus on developing and validating dispatch criteria that maintain high sensitivity for detecting severe injuries while improving specificity, particularly for vulnerable populations identified in our study.

## Data Availability

The data can be accessed through The Norwegian Trauma Registry and Oslo EMCC by application.

## Appendix

See Table

**Table 10:** Variable coverage

Variable	Number	Missing	Coverage (per cent)
Age	3633	0	100
Gender	3633	0	100
NISS	3384	249	93
Injury mechanism	3609	24	99
Prehospital GCS	3078	555	85
Centrality Index	3566	67	98

^10^.

## Abbreviations

EMCC: Emergency medical communication centre
EMS: Emergency medical services
NISS: New Injury Severity Score
ISS: Injury Severity Score
NTR: Norwegian Trauma Registry
TC: Trauma centre
ACTH: Acute care trauma hospital
GCS: Glasgow Coma Scale
CI: Centrality Index
SD: Standard deviation
IQR: Interquartile range
OR: Odds ratios
